# Triton, a new species-level database of Cenozoic planktonic foraminiferal occurrences

**DOI:** 10.1038/s41597-021-00942-7

**Published:** 2021-06-28

**Authors:** Isabel S. Fenton, Adam Woodhouse, Tracy Aze, David Lazarus, Johan Renaudie, Alexander M. Dunhill, Jeremy R. Young, Erin E. Saupe

**Affiliations:** 1 Department of Earth Sciences, University of Oxford, Oxford, UK; 2 School of Earth and Environment, University of Leeds, Leeds, UK; 3 Museum für Naturkunde, Leibniz-Institut für Evolutions- und Biodiversitätsforschung, Berlin, Germany; 4 Department of Earth Sciences, University College London, London, UK

**Keywords:** Palaeontology, Palaeoecology, Macroecology

## Abstract

Planktonic foraminifera are a major constituent of ocean floor sediments, and thus have one of the most complete fossil records of any organism. Expeditions to sample these sediments have produced large amounts of spatiotemporal occurrence records throughout the Cenozoic, but no single source exists to house these data. We have therefore created a comprehensive dataset that integrates numerous sources for spatiotemporal records of planktonic foraminifera. This new dataset, Triton, contains >500,000 records and is four times larger than the previous largest database, Neptune. To ensure comparability among data sources, we have cleaned all records using a unified set of taxonomic concepts and have converted age data to the GTS 2020 timescale. Where ages were not absolute (e.g. based on biostratigraphic or magnetostratigraphic zones), we have used generalised additive models to produce continuous estimates. This dataset is an excellent resource for macroecological and macroevolutionary studies, particularly for investigating how species responded to past climatic changes.

## Abstract (summary)

**Table:** 

Measurement(s)	Abundance • age estimate • paleocoordinate
Technology Type(s)	digital curation
Sample Characteristic - Organism	Foraminifera
Sample Characteristic - Environment	deep marine sediment

Machine-accessible metadata file describing the reported data: 10.6084/m9.figshare.14655564

## Background & Summary

Planktonic foraminifera are unicellular zooplankton found throughout the world’s oceans. They have calcareous shells or ‘tests’ with morphological variation that allows for specimens to be identified to species level. The morphological species concepts used to identify foraminifera species based on test characteristics agree approximately with genetic species concepts^1–3^, with the level of cryptic speciation seemingly no more frequent than in other groups e.g. Agapow, *et al*.^4^. On death, many of their calcareous tests are deposited on the ocean floor, where they contribute – often in significant amounts – to the sediment^5^. Consequently, planktonic foraminifera have one of the most complete species-level fossil records of any group^6^.

The fossil record of planktonic foraminifera has been used to study fundamental evolutionary and ecological questions, such as the relative role of abiotic versus biotic drivers in clade diversification^7^, the temporal persistence of large-scale ecological patterns such as the latitudinal biodiversity gradient^8^, the importance of fossils for understanding diversity dynamics^9^, and the potential of ancient extinction events to inform conservation today^10^. Our new database of planktonic foraminifera occurrence data aims to broaden the potential and increase the accuracy of analyses that address these and other key ecological and evolutionary questions.

Over the past 50 years, a series of international projects have sampled seafloor and sub-seafloor sediments, including the Deep Sea Drilling Project (1968–1983), the Ocean Drilling Program (1985–2004), the Integrated Ocean Drilling Program (2004–2013) and the International Ocean Discovery Program (2013–2023). Of 375 expeditions, 158 have published species-level data on planktonic foraminifera, whether for biostratigraphic purposes or community analyses as part of results from cruise activity (e.g. Saffer, *et al*.^11^, Tamura, *et al*.^12^) or later reanalyses of the data, producing a wealth of palaeobiological data^13^. Many smaller, primarily piston or drop-core expeditions have also produced useful species-level data on planktonic foraminifera. However, no single, easily accessible resource exists that documents spatiotemporal records from these sources. Previous databases of planktonic foraminifera either contain only a subset of existing samples (e.g. Neptune^14–16^), or hold data in an archive structure, consisting of many separate datasets in different formats e.g. PANGAEA^17^.

**Fig. 1:**
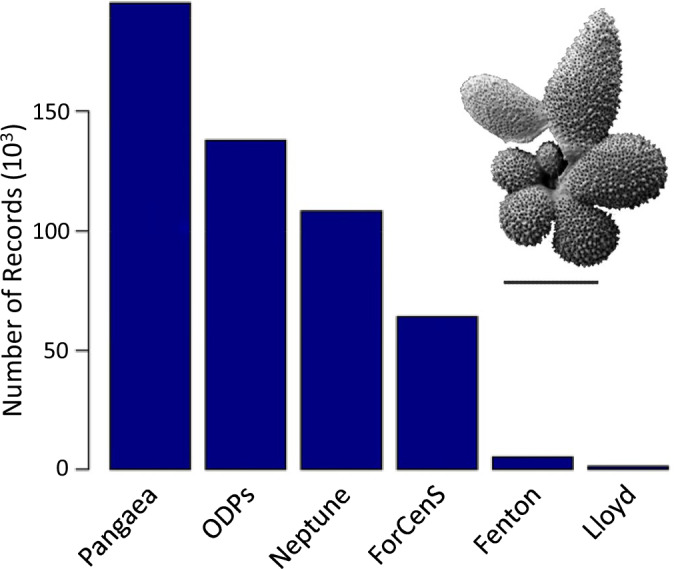
The number of records (in 1000 s) from each of the different data sources in Triton. The referenced data sources are: Pangaea^17^, ODPs (ocean drilling projects: DSDP, ODP and IODP), Neptune^14–16^, ForCenS^21^, Fenton, *et al*.^8^, Lloyd, *et al*.^22^. Inset: *Globigerinella adamsi*, an example planktonic foraminiferal specimen from the Paleogene GLObal Warming events “GLOW” cruise^44^, Southwest Indian Ocean, scale bar = 200 μm.

Here we describe the creation of Triton, a species-level occurrence based dataset that brings together planktonic foraminiferal sediment data for the Cenozoic from multiple sources (Fig. 1). The data are curated to ensure consistency in metadata across different sources. The taxonomy is updated to ensure consistency with publications of the Paleogene and Neogene Planktonic Foraminifera Working Groups (see “PFdata” in the figshare data repository^18^). Age models are similarly updated to the GTS 2020 timescale^19^, and, where necessary, more precise age models are calculated. Paleo-coordinates of fossil samples are estimated using a single plate rotation model. The new methods developed for Triton and described in this paper can be applied across sediment samples for other fossil groups.

**Fig. 2:**
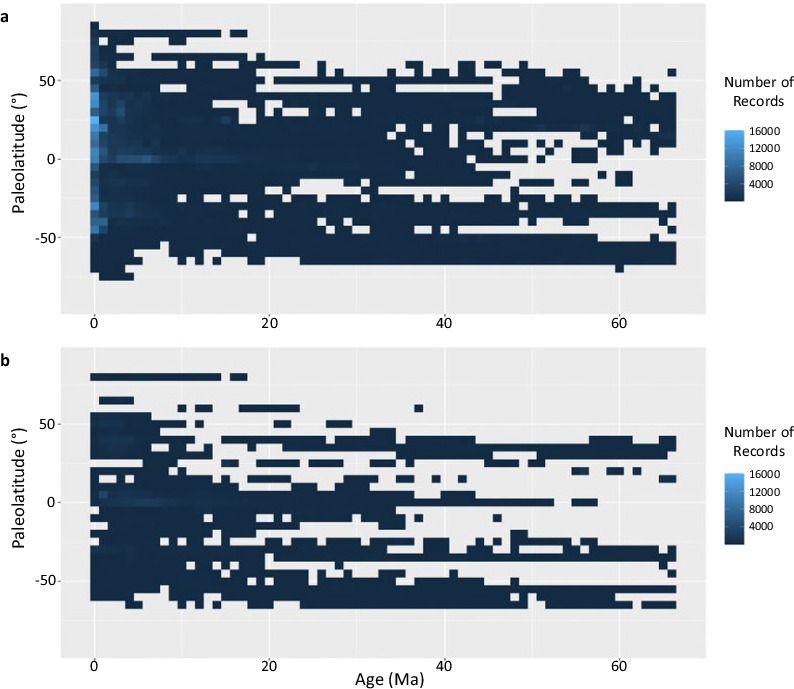
The latitudinal spread of data through time, showing (**a**) Triton and (**b**) the current data in Neptune. The squares are coloured to show the number of records, where a record is a row in the database (i.e. a species at a given location for a given age).

**Fig. 3:**
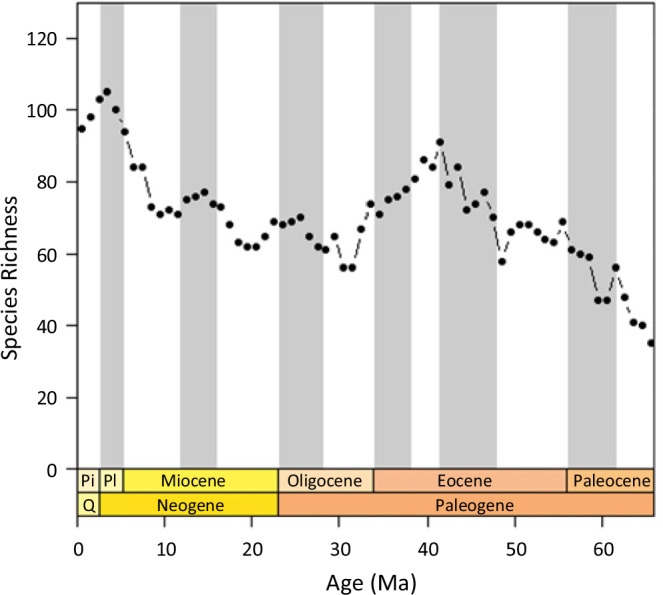
Species richness through time estimated from Triton (i.e. the number of species in each 1 Ma time bin). The pattern observed in Triton matches our understanding of diversity through the Cenozoic, particularly capturing the extinctions that occur at the end of the Eocene at 34 Ma^7^. The vertical lines indicate geological stages. Pl – Pliocene; Pi – Pleistocene. Note this plot uses the trimmed version of the data.

Triton provides a single access point for comprehensive spatio-temporal planktonic foraminifera data across the Cenozoic. It contains four times as many records as the previous largest planktonic foraminiferal distribution database, Neptune, and has a more comprehensive latitudinal spread through time (Table 1, Fig. 2). Diversity curves through time plotted from the Triton data indicate major features of species richness changes in planktonic foraminifera (Fig. 3), such as the end Eocene extinctions at 34 Ma^7,20^. These raw diversity curves depict a number of macroevolutionary events that were only apparent in Neptune once subsampling methodologies were applied, suggesting Triton has significantly more complete sampling. The Triton database offers many new opportunities for the use of planktonic foraminifera for a broad range of global studies or regional studies. These could be of a biological focus, based on ecological, evolutionary or conservation questions, for example investigating the past responses of planktonic foraminifera to drivers such as climate over a range of timescales. Alternatively, they could be geochemical questions, such as which cores contain abundant records of particular species for isotopic analyses, or oceanographic studies, investigating changes in ocean circulation or upwelling through time as a result of climatic or tectonic changes.

**Table 1:** Summary statistics showing the data spread improvement of Triton compared to Neptune, the previous largest compilation of foraminiferal data.

		Neptune	Triton	Percent Increase
**Total**		112,598	512,922	356%
**Species**	Macroperforate	102,466	474,876	363%
	Microperforate	10,132	38,046	276%
**Period**	Neogene	85,550	442,573	417%
	Paleogene	27,048	70,349	160%
**Latitude**	0–30°	60,315	297,135	393%
	30–60°	42,078	194,455	362%
	60–90°	4,231	21,332	404%
**Abundance**	Quantitative	8,899	229,045	2474%
	Semi-quantitative	82,752	228,802	176%
	Presence/absence	20,947	55,075	163%

## Methods

### Data sources

No single comprehensive dataset of planktonic foraminiferal distributional records currently exists. Instead, these data are available from a wide range of sources in many different structures. Some of these sources are compilations of existing data (e.g., Neptune^14–16^, ForCenS^21^), and others derive from individual sampling sites (e.g. ocean drilling expeditions). Triton combines these disparate sources (Fig. 1) to produce a single spatio-temporal dataset of Cenozoic planktonic foraminifera with updated and consistent taxonomy, age models, and paleo-coordinates.

Neptune is currently the most comprehensive database of fossil plankton data, with records exclusively from the DSDP, ODP and IODP representing planktonic foraminifera, calcareous nannofossils, diatoms, radiolaria and dinoflagellates^14–16^. A subset of these sites is included in Neptune, representing those with the most continuous sampling through time. The raw data from Neptune form the core of our dataset. All foraminiferal occurrences for the Cenozoic (i.e. last 66 Ma) were downloaded using the GTS 2012 timescale. In the download options, all questionable identifications and invalid taxa were removed, as were records that had been identified as reworked.

In addition to Neptune, three other compilation datasets were included in Triton: ForCenS^21^, which consists of global core-top samples; the Eocene data from Fenton, *et al*.^8^ created based on literature searches for planktonic foraminiferal data in the Eocene; and the land-based records from Lloyd, *et al*.^22^ that were created from literature searches. The marine records in Lloyd, *et al*.^22^ were not included, as they were obtained from Neptune.

Following preliminary compilation of existing datasets, we identified all legacy DSDP, ODP and IODP cores missing from Triton. The online DESCLogik (http://web.iodp.tamu.edu/DESCReport/) and Pangaea^17^ databases were then mined for .csv files containing planktonic foraminiferal species count data for the missing cores, supplemented with data from AWI_Paleo (URI: http://www.awi.de/en/science/geosciences/marine-geology.html), GIK/IFG (URI: http://www.ifg.uni-kiel.de/), MARUM (URI: https://www.marum.de/index.html), and QUEEN (URI: http://ipt.vliz.be/eurobis/resource?r=pangaea_2747). All additional cores were assessed individually by inspecting the scientific drilling proceedings to determine whether sites were suitable to contribute to our dataset. The primary assessment criterion was identification of continuous sedimentary sections, wherein two or more confidently assigned consecutive chronostratigraphic tie points existed to allow for construction of age models.

In addition to these longer cores, many sediment sampling projects have produced planktonic foraminiferal distribution data from shorter cores that tend to correspond in age to the last few million years. The website PANGAEA^17^ (www.pangaea.de) has been used as a repository for most of these occurrence data. This website was searched using the terms “plank* AND foram”, with resulting datasets downloaded using the R package ‘pangaear’^23^. These datasets were filtered to exclude records collected using multinets, sediment traps or box cores, as these methods produce samples not easily correlated to sediment cores. Column names allowed for further filtering to exclude records with no species-level data, records that had only isotopic data (rather than abundance data), or records with no age controls.

### Data processing

The data sources underpinning Triton serve their records in different formats. Therefore, processing was necessary to convert records into a unified framework, with one species per row for each sample and associated metadata (see below for details). Some metadata could be used without modification when available (e.g. water depth, data source), whereas other data needed processing to ensure consistency (e.g. abundance, paleo-coordinates, age). Without this processing, samples from different sources were not directly comparable. Where data were not available, they were set to NA. Those records with missing data in crucial columns (species name, abundance, age, and paleo-coordinates) were removed from the final dataset. All data processing was performed using R v. 3.6.1^24^.

Taxonomic consistency is essential to enable comparison of datasets created at different times. The species and synonymy lists used in Triton are based on the Paleogene Atlases^20,25,26^, with additional information from mikrotax^27^ (http://www.mikrotax.org/pforams/). These sources were supplemented, when necessary with more up to date literature including Poole and Wade^28^ and Lam and Leckie^29^. (A full list of the taxonomic sources can be found in the PFdata.xlsx file^18^.) A synonymy list was generated to convert species names to the senior synonym. At the same time, typographic errors were corrected. For example, *Globototalia flexuosa* should be *Globorotalia flexuosa*. Exclusively Mesozoic taxa were omitted, as were all instances when species names were unclear or imprecise (i.e. not at the species level). Junior synonyms were merged with their senior synonyms and their abundances summed, although the original names and abundances are also retained in the processed dataset. For presence/absence samples, these numerical merged abundances were set to one (i.e. present). The full species list and list of synonyms can be found in the accompanying data.

Abundance data for planktonic foraminifera are provided in different formats: presence/absence, binned abundance, relative abundance, species counts, and number of specimens per gram. These metrics were converted into numeric relative abundance to make comparisons easier, although both the original abundance value and its numeric version are retained, as is a record of the abundance type. Presence/absence data were converted to a binary format (one for present; zero for absent). Species counts were converted to relative percent abundances based on the total number of specimens in the sample (this was calculated where it was not already recorded). When full counts were not performed, binned abundances were frequently used. These binned abundances were converted into numeric abundances based on the sequence. So, for example, the categorical labels of N, P, R, F, C, A, D (indicating none, present, rare, few, common, abundant, dominant) were converted to a numerical sequence of 0 to 6. As the meaning of letters can depend on the context (e.g. ‘A’ could be absent or abundant), conversion was done in a semi-automated fashion on a sample-by-sample basis. A value of 0.01 was assigned to records where an inconsistent abundance was recorded (e.g. samples with mostly numeric counts but a few species were designated ‘P’, indicating presence). Samples with zero abundance were retained in the full dataset to provide an indication of sampling.

The age of samples were recorded in multiple ways. For some samples, age models provide precise numerical estimates of the age (e.g., those in Neptune). Other samples are dated relative to stratigraphic events such as biostratigraphic zones (including benthic and planktonic foraminifera, diatoms, radiolarians and nannofossils) or magnetic reversals. In this case, ages sometimes needed to be converted to reflect revised age estimates. The start and end dates of biostratigraphic zones are defined in relation to events in marker species, e.g. their speciation, extinction or acme events. All such marker events were updated to their most recent estimates and tuned to the GTS 2020 timescale^19^. The process of updating included correction of synonymies. Additional care was taken to ensure the correct interpretation of abbreviations (e.g. determining whether LO meant lowest occurrence or last occurrence) based on the entire list of events for a study. Where up-to-date ages were not available or events were ambiguous, they were removed from the age models.

The marker events defining a zone can depend on the zonal scheme used. For example, Berggren^30^ defined the base of the planktonic foraminifera zone M8 as the first occurrence of *Fohsella fohsi*. Wade *et al*.^31^ used this same event to define the base of M9. Therefore, the zonal scheme was recorded when collecting age models, to accurately convert ages to the GTS 2020 time scale. Some marker events have different ages depending on the ocean basin or latitude, and these differences are not necessarily well studied^31,32^. Where these differences in marker events have been recorded, the coordinates of a site were used to determine whether sites were in the Atlantic or Indo-Pacific Ocean, and whether they were tropical or temperate (with the division at 23.5° latitude). However, this is an area where more research is needed to improve the accuracy of higher-latitude dating^32^. Magnetostratigraphic ages were also tuned to the GTS 2020 timescale.

**Fig. 4:**
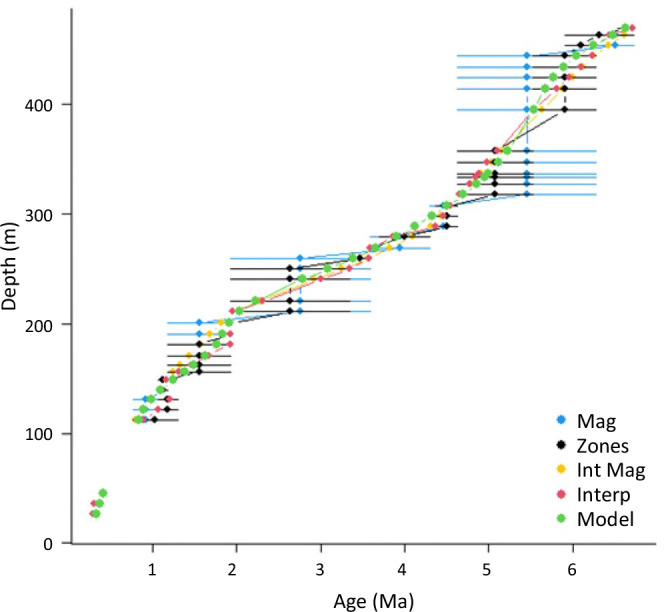
Different age model estimates applied to core material from IODP Site U1499A in the South China Sea. Mag – mean age based only on the magnetostratigraphic marker events. Zones – mean age based on all the marker events. Int Mag – interpolation of the points between the magnetostratigraphic marker events. Interp – interpolation between the full set of marker events. Model – the model of age as a function of depth. Note the hiatus between 50 and 100 m. For the shallower section of the dataset, with only three data points, a simple linear model was used. For the deeper section, a GAM smooth was fitted. For this site, the model predictions were chosen as the best fit.

We constructed new age models for samples not already assigned a numeric age. Where the depths of biostratigraphic events were already recorded, these were converted directly to GTS 2020. Where samples were not given any ages, often the case for the cores collected in the early days of ocean drilling, ages were reconstructed from the shipboard and post-cruise biostratigraphic data available in DESCLogik, Pangaea, and drilling publications. For holes where no tie point data were retrievable, biostratigraphic count data were extracted directly from drilling publications, and biostratigraphic events were assigned via GTS 2020. The first and last occurrences in raw shipboard biostratigraphic data often do not represent true datums, and careful assessment of the shipboard, and post-cruise literature was a prerequisite to confidently assigning chronostratigraphic datums. Tie point depths were assigned as the midpoint depth between the core sample before and after an event. For example, for an extinction event, the recorded depth was the midway point between the last recorded occurrence of a species and the first sample from which the species is absent. All sites were assessed individually to determine the age of the seafloor. Where IODP reports or sample-based publications strictly stated that the sediment surface (i.e. 0.00 meters below seafloor (mbsf)) was deemed to be “Holocene”, “Recent” or “Modern” in age, an additional 0 Ma tie point was assigned appropriately. All samples present outside the maximum/minimum age tie points for that site were removed, as they could not be confidently assigned an age. During assessment, individual drilling reports were investigated for geological structures. Where features such as unconformities, reverse faults, stratigraphic inversions, décollements, and major slips and slumps were identified, separate age models were generated for individual intact stratal intervals to account for potential externally emplaced or repeated strata (see “Age models” and “Triton working” in the figshare data repository^18^). Similarly, age gaps of greater than 10% of the age range of the core were classified as hiatuses, leading to separate age models (see Fig. 4). Cores of denser sediments that have been sampled using rotary drilling will often have only ~50–60% recovery in a core (9.5 m)^33^. As it is not possible to determine where the recovered core material came from within this length, all intact core pieces are grouped together as a continuous section from the section top, regardless of where the pieces were sourced (e.g. 4.5 m of recovered material will be recorded as 0–4.5 m of cored interval even if some came from 9–9.5 m). Consequently, age estimates within cores where recovery was low, typically the samples collected longer ago, will necessarily be less certain.

Using the updated marker event ages, we created age-depth plots and modelled the best fit to the data. There are different ways of creating these models, and multiple methods were applied to each core. The one that provided the best fit to the original data was chosen (the different age models are available in “Age models” in the figshare data repository^18^). These choices were confirmed manually (see Fig. 4). The simplest age model used interpolation of the marker events to create ‘zones’ and assign estimated ages assuming a continuous sedimentation rate between the start and end of each of these zones. Where the events do not provide a continuous sequence (e.g. gaps in the zonal markers), age estimates were assigned as the mean of that zone with error estimates of the width of the zone. Where magnetostratigraphic events were present they were given preference. This method leads to different estimates of sedimentation rate for each zone. The more complex age model estimates a smoother sedimentation rate. When there were fewer than 5 marker events, a linear model of age as a function of depth was fitted for the entire core. For larger datasets, generalised additive models (GAMs) for the same variables were used, to allow for variation in sedimentation rates through time. GAMs were run using the mgcv R library, with a gamma value of 1.1^34^. The type of age model used in the analysis was recorded. Where appropriate, the number of points and the r^2^ of the model are recorded to give an indication of the accuracy of the age model.

The latitude and longitude coordinates of samples were recorded in decimal degrees. For all samples except modern ones, plate tectonic reconstructions were necessary to determine the coordinates at which the sample was originally deposited. Reconstructions were performed using the Matthews, *et al*.^35^ plate motion model, which is an updated version of the Seton, *et al*.^36^ model used by Neptune. Comparisons of age models^35–39^ suggest this model is most appropriate for the deep sea environment where most of the samples occur, and is able to assign coordinates to significantly more sites than the Scotese^39^ GPlates model. This test was performed with a subset of the data (10633 unique sites); the Matthews, *et al*.^35^ model provided paleocoordinates for 95% of the data, whilst the GPlates model only provided coordinates for 17% of the data. The calculation of paleocoordinates was automated using an adaptation of https://github.com/macroecology/mapast.

When sediment samples are derived from multiple sources, duplication will inevitably occur. All such duplicated records, identified based on the combination of species, abundance, sample depth, and coordinate values, were removed. Additionally, working on an individual record level, species that occurred significantly outside their known ranges were flagged (following updated age models) on the assumption these records were misidentifications, contamination or re-working. Records were classified as falling significantly outside their known range if they were more than 5 Ma outside the species’ range in the Palaeogene (66-23 Ma) and more than 2 Ma in the Neogene (23-0 Ma). These values were chosen based on the tradeoff between removing reworked specimens and allowing for some errors in the age estimates. Age estimates for older samples tend to be less precise. Ages were obtained from Lamyman *et al*. (in prep) and are available in “PFdata” in the figshare data repository^18^. In total, 10,990 suspect records were flagged (~2% of all records).

## Data Records

The final dataset (“Triton” in the figshare data repository^18^) consists of one row per species for each sample depth from a core site. The associated metadata for these records can be categorised into a set of groups relating to the source of the data, the abundance of the species, the age of the sample, the geographic position of the site, the ocean drilling information (where appropriate), and the sampling procedures followed. These categories are explained in detail below.

The source of the data (**source**) is recorded based on the data citation and **year** in which it was collected. The primary data sources (e.g. Neptune, Pangaea) are given unique IDs (**db.source**). Individual datasets within this are given unique IDs (**db.ID**); these are particularly relevant for Pangaea where multiple, separate datasets exist. Each site is given a unique **holeID**, and samples within sites are designated using the **sampleID** (which is a unique number added to the **holeID**). The **rowID** is created by combining the database ID, the **sampleID**, and a unique number assigned to each row (i.e. species). The **person** who entered the data and **date** of the most recent update of that entry is also recorded.

The original species names assigned are listed in (**orig.species**). Where species were identified as synonyms and their records merged, both names are included in this column separated by a comma. The **species** column records the currently accepted name. Similarly, the original abundance column (**orig.abundance**) contains the abundance (or abundances for synonyms separated by commas) in its original form. The abundance units (**abun.units**: relative abundance, count, number per gram, binned, presence/absence) are recorded. **Abundance** is a numeric version of the abundance (**orig.abundance**). Where the number of counted individuals was recorded, it is provided in the **total.IDd** column. This total is also included in the number of individuals (**num.ind**) column with an estimated version of the total where it was not originally measured, which sums the numeric abundance of all species for each sample. The relative abundance (**rel.abun**) is then calculated using the estimated abundance divided by the total number of individuals.

For each individual sample, the **sample.depth** records the depth in the sediment from which the sample was taken; this is the mbsf (metres below sea floor depth) rather than the mcd (composite depth). The sample **age** provides the numeric age, whether from the original data or calculated using new age models, and the **age.err** indicates the precision of this estimate. The **segment** records where there were hiatuses in the sample, with separate age models being run for each segment. **Age.calc** indicates the type of age model used (orig, zone, magneto, interp, model). The age estimates from each of these different age models are also recorded separately. Original age, where the numeric age was already recorded, is indicated by an **age.calc** of “orig”, and no age model estimates. Zone ages were based on marker events both biostratigraphic and magnetostratigraphic. These are defined by the **zone**, with the **zon.age** being the mean of the **age.st** and **age.en**, and the range being given by **rng.age**. Interpolated ages (interp) use simple interpolations of these zonal markers by depth to give **int.age** and **err.int.age**. Where the models are based only on magnetostratigraphic age (magneto), the **mag.zone** indicates the markers, with the **mag.age** being the mean of **mag.age.st** and **mag.age.en**. The **int.mag.age** is the interpolation of these zonal markers, with the **err.int.mag.age** indicating the error in this estimate. The GAM (or linear model) estimate of the age is given by **mod.age**, with the r^2^ (**r2**) of the model, and the number of points (**n.pts**) it is based on giving an indication of accuracy. The **age.model** identifies the original age model used in the datasets, e.g. which biostratigraphic zonation was used. When the age was already numeric, this was designated GTS2012 (updated to GTS2020). The type of events used to determine the age were also recorded (**AM.type**). Visual representations of these age models are available in the “Age models” file in the figshare data repository^18^.

The **latitude** and **longitude** columns contain details about the sample site location, along with the current **water.depth** of the sample. Paleocoordinates (**pal.lat** and **pal.long**) were calculated using Matthews, *et al*.^35^. Where appropriate, ocean drilling program information, including the **leg**, **site**, **hole**, **core**, **section** and **sample.top** (in cm), were also recorded.

Differences in sampling strategy between sources introduces a possible source of bias. Therefore, where this information is available, sampling strategy was recorded. The **reason** indicates the purpose for which the data was originally collected: biostratigraphy, community analysis, proxies, selected species. This information will be useful for Triton users to determine whether the full dataset or only a subset is appropriate for their analysis. The **sample.type** indicates the method used to collect the sediment (e.g. piston core, box core). Sample **processing** details, when that information was available, records how the samples were processed, e.g. what sieve size was used, how many specimens were counted. The **preservation**, where it was recorded, gives an indication of whether thin walled species are likely to be absent. **Trim** gives an indication of whether the record falls significantly outside the known range of the species and therefore should be included (inc) or excluded (exc) if trimming is used. The cut-off for the Neogene is 2 Ma outside the species known range, and 5 Ma for the Palaeogene. These are likely to be taxonomic mis-identifications or the result of reworking.

The files and code required to run this dataset are provided in the figshare data repository^18^. The original stratigraphic events are provided in “Timescale conversion” and converted to the GTS 2020 dates using the “Ages” file. The species data is provided in “PF data”. The original datasets are in “Triton data”, and the intermediate stages of the data processing are provided in “Triton working”. The code to create the final dataset (“Triton”) is in the “Triton code”.zip file. The “Readme” file provides more details.

## Technical Validation

The final Triton dataset contains 512,922 non-zero records, spread throughout the Cenozoic (Table 1). Neptune, the previous most complete dataset, contains 112,598 records. To put our dataset in context, the largest macrofossil dataset at an equivalent taxonomic level are bivalves in the Palaeobiology Database, with 197,606 records for the entire Phanerozoic as of November 2020, and only 79,427 for the Cenozoic. The full Triton dataset, including all the records where a species’ abundance is recorded as zero, contains 1,716,087 records. These records derive from a range of sources (Fig. 1), which have different degrees of consistency in their structure and taxonomic data. For example, Pangaea data come from multiple different studies with many unique data structures.

Figure 2 shows how the spread of records varies through time and with latitude (see also Table 1). Records are most dense in more recent time intervals due to the challenges of coring deeper sediments^40,41^. Record gaps can also result from lack of preserved calcareous sediment due to dissolution. For example, in the modern ocean, the mid-latitude Pacific is particularly lacking in calcareous sediments as are Neogene high latitudes, since the older bottom water found there is more acidic^42^. Practical limitations of obtaining samples can also influence sampling in high latitudes, which typically require relatively calm oceans and ice-free conditions for most of a cruise, helping to explain the relative lack of records above 60 degrees. Our efforts in this paper are concentrated on compiling Cenozoic data, because they are more plentiful and widespread than older data.

The full species list of Cenozoic planktonic foraminifera is obtained from Lamyman *et al*. (in prep) and provided in “PFdata” in the figshare data repository^18^. When compared with this species list, our dataset contains records for 90% of valid species (394 of 438). Those species with no records tend to be recently identified (e.g. *Globoturborotalita paracancellata* which was described in Wade, *et al*.^26^), rarely used (e.g. *Turborotalia altispiroides* which was described in Bermúdez^43^) or actually rare (e.g. *Protentelloides dalhousiei*). For those species where there is at least one record, we can estimate the completeness of that record based on the fraction of age bins in which a species occurs between its speciation and extinction; 100% completeness implies that a species is found in every time bin of its expected range. Using 1 Ma bins, the median completeness for species is calculated to be 100% – 240 species (62.5%) having a ‘complete’ fossil record at that resolution. The mean completeness at this resolution is 87.8%. At the finer resolution of 0.5 Ma bins, 198 species (51.6%) have 100% completeness, while the mean is 83.4%. With this relatively high completeness level, plots of diversity through time (Fig. 3) indicate many of the major features of diversity change, such as the end Eocene extinctions at 34 Ma, are identified.

## Usage Notes

The main version of the Triton dataset contains only the positive abundances. There is also a column (trim) that indicates whether species fall significantly outside their known ranges, which can be used to produce a trimmed dataset. However, this could potentially remove some samples that are true presences rather than reworking as a result of inaccurate speciation or extinction dates. As how to define the cut-off is a personal decision, the untrimmed version of the dataset is provided here. Additionally, speciation and extinction estimates might not be representative of the whole of a species’ geographical range, with many of these estimated based on subtropical zonal schemes (e.g. Wade, *et al*.^31^). Consequently, trimming is likely to be less precise for higher latitude sites, where regional speciation and extinction ages may differ, and the zonal age estimates themselves may be less accurate.

A dataset version including absences is also supplied, which has the potential to provide more detailed information about species distributions. However, the significance of absences depends on the scoring procedure of the original database. When foraminiferal distribution data is collected for community analyses, recording prescribed species abundances through time, species absence is more informative, than when foraminiferal analysis is focused only on biostratigraphic marker species. In the first case if a species was on the list and not recorded, it is likely it was searched for and not found, whereas in the second, an absence could imply it was not searched for. Absences for studies explicitly focussing on community analyses are more informative, and allow, for example, more precise studies of species climatological preferences.

Similarly, we urge caution when using this data for site level diversity studies. Where planktonic foraminifera were identified for purely biostratigraphic purposes, only a subset of species may have been searched for or studied. If those species lists/records are taken at face value, estimates of alpha diversity will be artificially low. Consequently, alpha diversity estimates should only be taken from studies (approximately 40% of the total dataset) that explicitly recorded the entire community. More comprehensive studies of diversity should focus on gamma diversity using binned ages and should account for spatial coverage.

The Triton dataset is provided as an open access resource with this paper^18^. When using it, we ask that you cite this paper. If a significant fraction of your data subset derives from ForCenS^21^ or Neptune^14–16^, we ask those papers are also cited.

Although this analysis aimed to target all the larger online sources of data for Triton there are inevitably some datasets which are not included, such as data tables from individual journal articles. By including the code used to reformat the Triton dataset, we aim to make sure that these datasets, and future research, can be added for further analyses. Additionally updates to the ages of marker events or the taxonomy can be incorporated into Triton. As an example of how to update the taxonomy, we have included the updates from Lam and Leckie^29^ and Lam and Leckie^32^ (which are incorporated into Triton) as a separate file (Updated Taxonomy.xlsx^18^), and provide the code (Triton_Update.R^18^) to make these, or future, changes.

## Data Availability

All the code used to generate this database is available in the TritonDB repository on github (https://github.com/IFenton/TritonDB), as well as in “Triton code” in the figshare data repository^18^.
