# Correction: Triton, a new species-level database of Cenozoic planktonic foraminiferal occurrences

**DOI:** 10.1038/s41597-025-05704-3

**Published:** 2025-08-08

**Authors:** Isabel S. Fenton, Adam Woodhouse, Tracy Aze, David Lazarus, Johan Renaudie, Alexander M. Dunhill, Jeremy R. Young, Erin E. Saupe

**Affiliations:** 1 Department of Earth Sciences, University of Oxford, Oxford, UK; 2 School of Earth and Environment, University of Leeds, Leeds, UK; 3 Museum für Naturkunde, Leibniz-Institut für Evolutions- und Biodiversitätsforschung, Berlin, Germany; 4 Department of Earth Sciences, University college London, London, UK

Correction to: *Scientific Data* 10.1038/s41597-021-00942-7, published online 28 June 2021

In this article Isabel S. Fenton and Adam Woodhouse should have been denoted as equally contributing authors. The original article has been corrected.

